# A Survey of the Multi-Sensor Fusion Object Detection Task in Autonomous Driving

**DOI:** 10.3390/s25092794

**Published:** 2025-04-29

**Authors:** Hai Wang, Junhao Liu, Haoran Dong, Zheng Shao

**Affiliations:** School of Automotive and Traffic Engineering, Jiangsu University, Zhenjiang 212013, China; liujunhao77777@163.com (J.L.); 16605358877@163.com (H.D.); shaozheng77777@163.com (Z.S.)

**Keywords:** multi-sensor fusion, object detection, LiDAR, cameras, environmental perception

## Abstract

Multi-sensor fusion object detection is an advanced method that improves object recognition and tracking accuracy by integrating data from different types of sensors. As it can overcome the limitations of a single sensor in complex environments, the method has been widely applied in fields such as autonomous driving, intelligent monitoring, robot navigation, drone flight and so on. In the field of autonomous driving, multi-sensor fusion object detection has become a hot research topic. To further explore the future development trends of multi-sensor fusion object detection, we introduce the mainstream framework Transformer model of the multi-sensor fusion object detection algorithm, and we also provide a comprehensive summary of the feature fusion algorithms used in multi-sensor fusion object detection, specifically focusing on the fusion of camera and LiDAR data. This article provides an overview of feature fusion’s development into feature-level fusion and proposal-level fusion, and it specifically reviews multiple related algorithms. We discuss the application of current multi-sensor object detection algorithms. In the future, with the continuous advancement of sensor technology and the development of artificial intelligence algorithms, multi-sensor fusion object detection will show great potential in more fields.

## 1. Introduction

Object detection refers to a technology in the field of computer vision that aims to identify the category and location of specific objects in images and videos [1]. It has a wide range of applications in areas such as autonomous driving, safety monitoring, medical image analysis, industrial automation, drone applications, AR and VR, human–computer interaction, sports analysis and so on, and in many industries, it is driving the development of intelligence and automation. The quality of object detection directly affects the performance of high-level tasks such as object tracking, action recognition, and behavior understanding [2]. Therefore, object detection has always been a research focus in the field of vision.

Object detection can be divided into visual object detection, LiDAR object detection, and multi-sensor fusion object detection [3]. Visual object detection refers to the method of using image data obtained from a single camera or multiple cameras for object recognition and localization [4]. Currently, mainstream visual object detection can be divided into two categories: methods based on region, such as R-CNN [5], Fast R-CNN [6], Faster R-CNN [7], etc., which generate candidate regions for object detection, and methods based on single-stage detectors, such as the YOLO series [8], which directly detect the entire image. Compared to other methods, visual object detection has the advantages of low cost and rich color, texture, text, and shape information. However, due to its dependence on the environment, it is greatly affected by factors such as lighting, weather, and occlusion, and it also suffers from poor real-time performance [9]. LiDAR object detection refers to a technology that relies solely on point cloud data obtained by LiDAR for target recognition and localization [10].Mainstream LiDAR object detection can also be divided into two categories: methods based on clustering, such as DBSCAN [11], which group point clouds and identify independent targets through clustering algorithms, and methods based on deep learning, such as Point Net [12] and Point RCNN [13], which directly process point clouds and use neural networks to automatically learn features for object detection. LiDAR object detection has advantages such as high precision and high environmental adaptability [14], but it also has problems such as the difficult data processing and high cost.

Multi-sensor fusion object detection refers to the method of fusing data from multiple sensors, such as laser radar, cameras, millimeter-wave radar, and ultrasonic sensors, to identify, locate, and track targets [15]. Multi-sensor fusion object detection can improve robustness, reduce false positives and false negatives, and enhance environmental understanding, but it can also cause problems such as data asynchrony, data redundancy, conflicts, and computational complexity due to too many data sources [16]. However, these problems have been well addressed with the improvement of hardware and computational methods. Multi-sensor fusion can be divided into pre fusion, post fusion and feature fusion [17]. Pre fusion refers to the direct combination of original data from different sensors (images, point clouds) at the early stage of data fusion for unified processing. Post fusion refers to the process of independently processing and obtaining results from various sensors, and then integrating those results [18]. Feature fusion refers to the method of integrating features from different sensors at the feature extraction stage. Compared with pre fusion and post fusion, feature fusion has the advantages of high real-time performance, making full use of data, and effectively improving detection performance. It is the mainstream of multi-sensor fusion object detection at present [19]. This article summarizes the classic model architectures in sensor fusion object detection and some model architectures that perform well in terms of the accuracy and real-time performance in the field of autonomous driving.

In this review, we aim to summarize the current state-of-the-art LiDAR and camera fusion-based object detection algorithms in the field of autonomous driving. The article is organized as follows. Section 2 introduces the origin of multi-sensor object detection. Section 3 provides an overview of three primary sensors: cameras, LiDAR, and millimeter-wave radar. Section 4 categorizes the camera and LiDAR object detection algorithms into feature-level fusion and proposal-level fusion, detailing specific object detection algorithms. Section 5 discusses commonly used datasets and evaluation metrics. Section 6 explores multi-task applications of multi-sensor fusion object detection algorithms, including segmentation and tracking. Finally, Section 7 concludes the article and offers recommendations for future developments in fusion-based detection algorithms.

## 2. The Origin and Development of Multi-Sensor Fusion Object Detection

In complex environments, a single sensor (such as a camera or LiDAR) often cannot provide sufficient information to accurately identify and locate targets [20]. Therefore, researchers have explored how to enhance the perception capability of the system by combining different types of sensor data. Early research on multi-sensor fusion focused primarily on mobile robots. Researchers used various sensors, such as ultrasound, infrared sensors, and cameras, for data fusion to improve the navigation and obstacle avoidance capabilities of robots [21]. With the development of computer vision and pattern recognition technologies, especially the advent of deep learning, the performance of object detection has significantly improved, providing new opportunities for multi-sensor fusion. Currently, multi-sensor object detection algorithms are applied in fields such as autonomous driving, drones, and agricultural engineering [22,23,24].

Initially, multi-sensor fusion relied on classical data fusion methods such as Kalman filtering and particle filtering. These methods were used for state estimation, but they were typically only applicable to linear systems and were sensitive to noise and uncertainty [25]. In the field of mobile robots, efforts were made to integrate data from LiDAR and ultrasonic sensors to improve the environmental perception capabilities of robots. At that time, the research mainly focused on basic sensor combinations and simple fusion algorithms. Subsequently, with the development of statistical learning and nonlinear filtering methods, the algorithms for multi-sensor fusion became more complex. For example, methods such as the extended Kalman filter (EKF) and unscented Kalman filter (UKF) were developed to handle nonlinear systems and more complex dynamic models [26]. The rapid advancement of deep learning technology further enhanced the feature extraction and pattern recognition capabilities, driving progress in multi-sensor fusion object detection. Researchers began using convolutional neural networks (CNNs) [27] to process image data and integrate it with data from other sensors. As a result, multi-sensor fusion technology has been widely adopted in fields such as autonomous driving, intelligent monitoring, and industrial and agricultural automation [28,29], accelerating the development of related technologies. In recent years, many deep learning algorithms based on multi-sensor fusion algorithms have emerged, which can directly process point cloud data and perform object detection, greatly improving the detection accuracy. With the improvement of computing power, real-time multi-sensor fusion has become possible [30]. Future research will focus on how to adaptively adjust fusion strategies based on environmental changes and task requirements to improve the system flexibility and robustness. Meanwhile, advances in deep learning can further optimize the decision-making process in terms of multi-sensor fusion and improve the efficiency and accuracy of object detection [31]. Effectively integrating data from different modalities (such as images, point clouds, etc.) to improve the accuracy and speed of object detection remains a significant challenge.

## 3. Characteristics of Three Sensors

**Figure 1:**
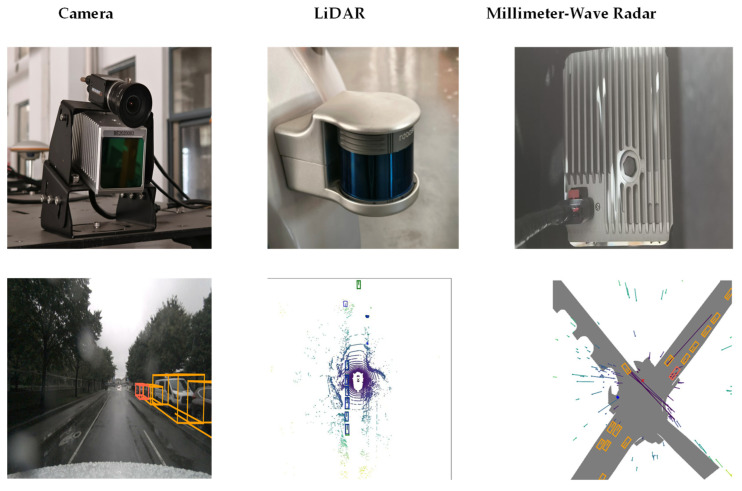
Multi-sensor appearance and detection performance comparison.

Autonomous vehicles predominantly use three types of sensors for object detection: cameras, MMW radar, and LiDAR. The appearance of these sensors and their respective detection effect diagrams are depicted in Figure 1. Each sensor exhibits distinct advantages and limitations in terms of the operational characteristics, as evidenced by their divergent performance metrics, as summarized in Table 1. The heterogeneous nature of sensor capabilities necessitates a multi-sensor fusion framework to synergistically enhance environmental perception across multiple dimensions, thereby ensuring robust safety assurance for both vehicle occupants and road users.

### 3.1. Camera

As one of the earliest sensors integrated into autonomous driving (AD) systems, cameras remain a cornerstone technology for manufacturers and researchers. They are primarily utilized for critical tasks such as object recognition, environmental mapping, lane detection, and target tracking. In recent years, deep learning (DL) [32] has achieved groundbreaking advancements in object recognition and tracking, leveraging large-scale datasets to learn robust feature representations, thereby replacing traditional methods reliant on handcrafted feature engineering. Building upon high-precision object recognition and tracking, autonomous systems can further execute advanced decision-making and control tasks, significantly enhancing the overall driving performance and safety.

Camera-captured images inherently project 3D spatial information into 2D representations. Camera calibration establishes the geometric relationship between pixel coordinates and real-world physical dimensions, enabling the extraction of target positions from images. Common calibration methods include optimization-based approaches, transformation matrix techniques, distributed calibration, Zhang’s calibration, and classical calibration frameworks [33]. Recent studies have explored the use of stereo or depth cameras to acquire depth-enhanced image data, which are then integrated into multi-sensor fusion algorithms via depth-aware calibration methods [34,35,36]. However, such approaches still exhibit significant gaps in the distance resolution compared to millimeter-wave radar (MMW radar) or LiDAR [37]. Furthermore, standalone camera systems face reliability challenges in adverse weather conditions (e.g., heavy rain, fog) or scenarios with abrupt illumination changes (e.g., tunnel exits) [38]. Compare to radar, cameras excel at capturing detailed contour, texture, and color distribution information of targets, facilitating accurate classification and recognition under non-extreme environmental conditions. Nevertheless, autonomous driving systems demand robust adaptability in all-weather environments and extreme scenarios, posing stringent requirements for camera-based perception systems.

### 3.2. LiDAR

As a critical sensor in autonomous driving systems, LiDAR has garnered significant attention due to its unparalleled 3D perception capabilities. It is primarily employed for environmental modeling, obstacle detection, target tracking, and high-definition map construction. Recent advancements in LiDAR technology, particularly in hardware performance and data processing algorithms, have markedly enhanced its operational efficacy [39]. By emitting laser beams and analyzing reflected signals, LiDAR generates high-resolution point cloud data, enabling precise 3D reconstruction of the surrounding environment. In autonomous driving systems, LiDAR’s high-fidelity perception supports robust path planning, decision-making, and control tasks, substantially improving the system safety and reliability.

LiDAR operates based on the time-of-flight (ToF) principle, calculating the target distances by measuring the round-trip time of the emitted laser pulses, thereby producing 3D point clouds. These point clouds not only provide positional information but also capture geometric shapes and surface details of objects. The calibration methodologies for LiDAR, including target-based calibration, motion-based approaches, and self-calibration techniques [40], are essential for ensuring measurement accuracy and system consistency. However, LiDAR’s performance may degrade under adverse weather conditions (e.g., rain, snow, fog), and its high manufacturing cost remains a barrier to widespread commercial adoption [41]. Compared to cameras, LiDAR’s key advantage lies in its ability to directly acquire high-precision 3D spatial data while remaining unaffected by ambient lighting variations, making it suitable for nighttime or rapidly changing illumination scenarios [42]. Nonetheless, LiDAR exhibits limitations in terms of target classification and semantic understanding, often necessitating fusion with camera data for comprehensive environmental perception [43]. Additionally, the computational complexity of processing dense point cloud data imposes significant demands on system resources, posing challenges for real-time applications [44]. Ongoing advancements in LiDAR technology—such as solid-state LiDAR development and algorithm optimization—underscore its indispensable role in autonomous driving, particularly in high-precision localization and complex environment perception [45].

### 3.3. Millimeter-Wave Radar

As a pivotal sensor in autonomous driving systems, MMW radar plays an indispensable role due to its exceptional capabilities in terms of ranging, velocity measurement, and all-weather environmental adaptability. It is primarily deployed for critical tasks such as obstacle detection, target tracking, speed estimation, and dynamic environment perception. Recent advancements in MMW radar technology, particularly in hardware enhancements and data processing algorithm optimization, have significantly improved its performance [46]. By emitting millimeter-wave signals and analyzing their reflection properties, MMW radar accurately captures the target distance, velocity, and azimuth information, providing highly reliable environmental perception data for autonomous systems. In complex dynamic scenarios, the real-time responsiveness and robustness of MMW radar ensure dependable support for path planning, decision-making, and control tasks.

MMW radar operates based on the transmission and reception of high-frequency electromagnetic waves. The output data not only include positional and velocity information but also effectively distinguish target motion states (e.g., stationary or moving). To enhance the measurement precision and system consistency, the calibration methodologies for MMW radar encompass target-based calibration, signal-processing optimization techniques, and multi-sensor joint calibration frameworks [47]. However, MMW radar exhibits limitations in relation to spatial resolution, struggling to capture fine geometric details of targets, and demonstrates reduced efficacy in detecting static objects [48]. Compared to LiDAR and cameras, MMW radar’s distinct advantages lie in its superior resilience against adverse weather conditions (e.g., rain, snow, fog) and complete insensitivity to illumination variations, making it an ideal choice for all-weather environmental perception [49]. Nevertheless, its limited capability in terms of target classification and semantic understanding often necessitate fusion with camera or LiDAR data to achieve comprehensive scene interpretation [50]. Furthermore, the computational complexity of processing multi-target scenarios imposes substantial demands on computational resources, presenting a persistent challenge for practical implementations [51].

Despite these challenges, continuous advancements in MMW radar technology—including hardware miniaturization and algorithm innovations—underscore its promising future in autonomous driving. Its unique strengths in terms of all-weather perception, dynamic target tracking, and cost-effective solutions position it as a critical enabler of scalable AD deployments [52]. Through deep integration with complementary sensors (e.g., cameras, LiDAR), MMW radar can further enhance the perception accuracy and system robustness, paving the way for higher levels of autonomous driving capabilities.

The field of perception systems primarily employs cameras, LiDAR, and millimeter-wave radar. Currently, the mainstream object detection approaches can be categorized into (1) camera-only detection, (2) LiDAR-only detection, (3) camera–LiDAR fusion detection, and (4) multimodal detection combining cameras, LiDAR, and millimeter-wave radar. Among these, camera–LiDAR fusion detection and vision-only detection have demonstrated superior performance in terms of both the accuracy and real-time capability. This article primarily focuses on reviewing camera–LiDAR fusion detection algorithms, as they represent a promising direction for autonomous driving perception systems. The rationale for this focus includes the performance superiority, sensor complementarity, and practical applicability. The following sections will provide a comprehensive taxonomy and analysis of the state-of-the-art camera–LiDAR fusion algorithms, examining their architectural designs, fusion strategies, and performance characteristics.

## 4. Multi-Sensor Feature Fusion Object Detection

**Figure 2:**
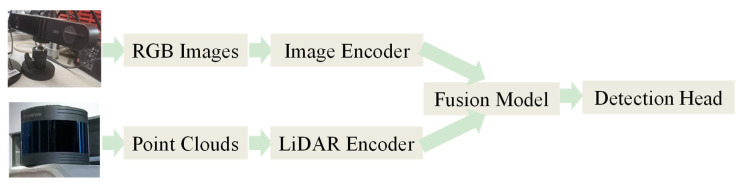
Multi-sensor object detection feature fusion workflow diagram.

The multi-sensor feature fusion object detection algorithm framework is shown in Figure 2. At present, mainstream multi-sensor object detection mainly utilizes the Transformer framework, a deep learning model designed to process sequence data. The Transformer was first introduced by Vaswani et al. [53] in 2017. The paper “Attention is All You Need” [53] introduces the Transformer architecture, which consists of two main components: the encoder and the decoder. The encoder and decoder incorporate several key components, such as the self-attention mechanism, multi-head attention, positional encoding, feedforward neural networks, layer normalization, and residual connections. This approach effectively reduces computational requirements while maintaining accuracy, thereby enhancing the real-time performance of the model [54]. Due to the flexible nature of this architecture, encoders and decoders can be disassembled or modified, leading to various adaptations of the Transformer structure and the emergence of numerous new architectures [55,56]. These novel architectures enable the overall framework to more comprehensively utilize information from both images and LiDAR point clouds. Additionally, they significantly enhance the real-time performance.

Compared to traditional CNN architectures, the self-attention mechanism proposed in Transformer models directly computes the relationships between any two positions, overcoming the limitations of local receptive fields inherent in CNNs. This makes Transformers particularly suitable for segmentation tasks and long-sequence data processing. Additionally, the parallelizable nature of self-attention significantly improves the training efficiency compared to RNNs. The cross-attention mechanism in Transformers further enables the natural fusion of multimodal data, substantially enhancing the framework’s flexibility for diverse applications. In the field of object detection, the Facebook team introduced an end-to-end object detection framework based on the Transformer architecture in 2020—DETR (DEtection TRansformer) [57]. This work represents a groundbreaking application of a Transformer in image-based object detection, significantly streamlining the traditional detection framework. The DETR framework comprises a convolutional neural network (CNN) backbone, a Transformer-based encoder–decoder structure, and a feedforward neural network (FFN) prediction layer. The appearance of DETR has catalyzed extensive subsequent research on Transformer-based object detection, including optimizations of the DETR framework, the adoption of more efficient computational approaches, and the integration of complementary techniques (e.g., convolutional layers) to improve performance. However, DETR also reflects some limitations of the Transformer structure, such as the high computational complexity, difficulty in processing super long sequences, strong data dependence, and need for large-scale data to leverage its advantages, but the advent of DETR has provided a conceptual foundation for the development of multi-sensor object detection models, marking a pivotal transformation in the field. It not only introduces a novel paradigm and methodology but also propels the field toward enhanced efficiency and accuracy [58]. At present, although there are various problems in the Transformer architecture, its application in the field of multi-sensor fusion still shows explosive growth, which may be because the Transformer fundamentally changes the fusion paradigm of multimodal data and breaks through the limitations of traditional methods.

### 4.1. Feature-Level Fusion

Feature-level fusion involves constructing a unified feature space (typically in the LiDAR space), where features from different modalities are extracted to form a multimodal feature volume. DeepFusion [59] implicitly completes multimodal alignment through learnable lightweight attention, avoiding the calibration error problem of traditional geometric methods (such as ICP [60]), but it lacks the display modeling of dynamic objects. In contrast, BEVFusion [61] constructs a unified Bev space through geometric projection, which retains a more stable spatial structure but requires additional view transformation calculations, which increases the amount of calculation and reduces the real-time performance. On the basis of BEVFusion, BEVusion4D [62] further introduced the LiDAR-guided view Transformer to improve the alignment accuracy by using time sequence information, while SimpleBEV [63] achieved a balance in real-time performance by simplifying the depth estimation process. FusionFormer [64] innovatively improved the encoder of the Transformer. Through the cross-attention and timing fusion module, it enhanced the multimodal interaction and improved the context understanding ability by using the historical BEV feature. This design not only maintains the structural advantages of the BEV space but also improves the adaptability of dynamic scenes. The cross-modal Transformer (CMT) [65] has a different approach. Instead of building a unified feature space, it uses a unified position-guided attention feature aggregation method to query image and point cloud features. This method further optimizes the computational efficiency while maintaining the same accuracy as FusionFormer, and it provides a new idea for lightweight deployment.

DeepFusion: The InverseAug and LearnableAlign techniques proposed in DeepFusion efficiently achieve image point cloud alignment through lightweight cross-attention, but their dependence on end-to-end training may limit their deployment on resource-constrained devices (such as edge computing). In addition, compared with the methods based on geometric alignment (such as BEVFusion), the robustness of LearnableAlign on dynamic objects (such as pedestrians) has not been fully verified. Although DeepFusion has high computational efficiency, its alignment accuracy may decline in long-distance scenes. Therefore, we recommend introducing a layered attention mechanism, low-resolution global alignment, and gradually refining local areas. DeepFusion can be easily applied to most 3D detection methods which are based on voxel as a plug-in, which is more suitable for industrial deployment but may sacrifice the potential of the deep fusion of cross-modal features. In subsequent studies, the LearnableAlign mechanism of DeepFusion may be considered to migrate to medical image fusion (such as ultrasound and MRI alignment), which would be affected by the small sample problem of medical data.

BEVFusion: BEVFusion creatively proposed a multimodal fusion paradigm based on the unified aerial view (BEV) representation space, and it realized the geometrically accurate alignment of camera and LiDAR features in the 3D space for the first time through the differentiable view transformation (LSS [66]). A full convolutional BEV encoder was designed to support multi-task learning such as detection and segmentation. In addition, SOTA (map 65.3%, NDS 70.2%) was achieved on the nuScenes [67] benchmark. However, this method has the limitation of increasing the projection error by 15–20% in dynamic scenes, which can be improved by adding timing information (such as BEVFusion4D and BEVDet4D [68]) or by adaptive grid sampling (such as EVT [69]). In addition, the balance between calculation and accuracy can be improved by optimizing the space (such as SimpleBEV) and extracting sparse BEV features. Moreover, BEVFusion performs poorly in extreme weather (such as dense fog).

BEVFusion4D: Based on BEVFusion, BEVFusion4D proposes a LiDAR-guided view Transformer (LGVT) By using LiDAR BEV as a spatial prior iteration to optimize the semantic query of camera BEV, it realizes cross-modal forward fusion under geometric constraints, alleviates the geometric dislocation of multi-view image projection, and improves the detection accuracy of occluded areas. In addition, its time warping alignment module (TDA) introduces deformable convolution to dynamically aggregate historical BEB frames, which solves the timing ambiguity caused by moving objects, and has better performance than the timing feature alignment module of BEVDet4D. The emergence of BEVFusion4D marks the key evolution of BEVFusion from static scenes to dynamic spatio-temporal modeling. However, there are still some problems with this method, such as the overdependence of LiDAR (17% reduction in long-distance performance on the nuScenes dataset), computational efficiency and low robustness (especially in extreme weather). In the future, the practicability can be further improved through lightweight design (such as sparse fusion), robustness enhancement (such as dynamic calibration) and long time-series expansion.

SimpleBEV: SimpleBEV is also optimized on the basis of the BEVFusion framework. It realizes depth estimation by camera through a cascade network and corrects it by using the depth information provided by the LiDAR point cloud, so as to improve the accuracy of depth estimation. In this method, the training reasoning decoupling mode utilization is proposed for the first time, which is an auxiliary branch using only camera BEV features introduced to make full use of camera information in the training phase, and the LiDAR feature extractor is improved by fusing multi-scale sparse convolution features. Experimental results show that SimpleBEV achieves 77.6% NDS accuracy on the nuScenes dataset, showing excellent 3D target detection performance. In addition, the design of SimpleBEV simplifies the multi-sensor fusion process, reduces the overhead of algorithm reasoning, and has good real-time performance. However, its performance still over-relies on the geometric truth of LiDAR. When extreme weather (such as heavy rain) leads to the decline of LiDAR performance, the camera branch performance is poor. Future work may include optimizing the depth estimation module and exploring more downstream applications.

FusionFormer: FusionFormer achieves efficient 3D target detection by improving the Transformer encoder. It uses the point cloud voxel cross-attention and image cross-attention modules, and it samples the original voxels and image features directly through the deformable attention mechanism, to avoid the loss of geometric information in the traditional BEV projection. The time fusion encoder dynamically aggregates the BEV features of the historical frames and significantly reduces the false alarm rate of the moving target. Moreover, the residual structure design ensures the stability in the absence of a single mode, and the accuracy is only reduced by 8% in extreme weather (such as heavy rain) scenarios. This suggests an idea for maintaining high accuracy in extreme weather. The framework supports the input of LiDAR features in the form of voxels or BEV, and experiments show that the voxel format retains the height information, which reduces the vehicle height prediction error by 15%, and is compatible with pure visual mode (NDS to 68.3% on dataset nuScenes). However, its real-time performance is also limited by deformable attention computation, and the FPS is only 4.0. In the future, sparse attention may be used to optimize real-time performance. FusionFormer provides an integrated paradigm of “fusion-timing fault-tolerance” for a multimodal Transformer. It provides a new idea for a follow-up study.

CMT: CMT innovatively proposes the implicit coordinate coding mechanism and the location-guided query generation strategy. It uses a multimodal coding module to avoid the geometric deviation of explicit view transformation, and it achieves 74.1% NDS (SOTA) on the nuScenes dataset. Compared with BEVFusion (explicit view transformation), CMT achieves the parallel interaction of multimodal tokens through coordinate coding, and the number of parameters is reduced by 15%, so that the detection speed can reach 6.0 FPS. Moreover, when it lacks LiDAR data, CMT can still maintain pure visual mode performance (NDS 68.3%). Recently, the IS-Fusion framework further improves the CMT performance to 72.8% through instance scene collaborative fusion, which seems to imply that pure coordinate coding may ignore high-level semantic associations. Moreover, the computational complexity of CMT still restricts the real-time performance.

Table 2 summarizes the advantages and limitations of the feature-level fusion algorithms. As illustrated, the strengths of the feature-level fusion algorithms include the following. (1) Information richness: By integrating data from multiple sources, these algorithms provide richer contextual information, thereby enhancing model performance. (2) Global feature modeling: They effectively capture relationships between different modalities, improving the understanding of complex scenes. (3) Reduced information loss: Fusion at the feature level preserves more original information, contributing to higher detection accuracy. However, the limitation include the following. (1) High computational complexity: The processing of higher-dimensional feature vectors may increase the computational overhead. (2) Implementation complexity: The processes of feature extraction and fusion can be intricate, requiring carefully designed network architectures. (3) Information redundancy: In some cases, different modalities may contain overlapping information, leading to feature redundancy. This redundancy can unnecessarily complicate the model without necessarily improving its performance. Therefore, a key challenge for future research into feature-level fusion is to efficiently select relevant information without losing critical data, reduce the model complexity, and minimize the computational costs while maintaining the accuracy. The development mainly focuses on improving the lightweight design of the feature selection (such as the hybrid upper and lower sampling module adopted by YOLOV10 [70] to reduce the parameters by 40% while maintaining the AP), adaptive fusion, cross-modal alignment, as well as intellectualization, enhancing the accuracy of the feature alignment, reducing the computational complexity and improving the interpretability of the model.

### 4.2. Proposal-Level Fusion

Proposal-level fusion methods leverage modality-specific proposals to maximize the utilization of multimodal data. F-PointNet [71], an early representative, relies on explicit geometric transformation to generate a 3D visual cone through a 2D detection frame to realize modal interaction, but it excessively relies on the display geometric transformation to increase the dynamic scene error by 15–20%. Full Sparse Fusion (FSF) [72] and SparseFusion [73] adopt a dual-mode instance generation strategy. Both of them are committed to promoting a completely sparse architecture to improve efficiency. However, the former has insufficient utilization of LiDAR information due to the camera-branch-dominated fusion, while the latter has lost the original perspective semantics through lightweight self-attention fusion features but forced alignment. The subsequent approach, MV2DFusion [74], enhances the computational efficiency by optimizing the trade-off between hierarchical attention accuracy and processing overhead. It improves the NDS to 76.7% on the nuScenes dataset through three innovations: the probability distribution of the modeling depth of uncertain perceptual queries, the hierarchical attention mechanism of the decoupled fusion decoder, and historical query enhancement, which significantly optimizes the performance of multimodal 3D detection. Different from them, Transfusion [75] achieves soft alignment between candidate frames and images through cross-attention. The AP of small target detection increases by 8.2%, and the pure visual mode maintains 68.3% NDS (in the nuScenes dataset). MV2DFusion and Transfusion both achieve implicit semantic fusion through the attention mechanism to improve the adaptability of dynamic scenes, but their performance still needs to be improved in extreme weather environments. The above-mentioned F-PointNet, FSF and SparseFusion all have the problem of modal deviation, and the subsequent research on the soft alignment of Transfusion and the decoupling fusion of MV2DFusion are all committed to solving this problem, and they all performed well.

F-PointNet: F-PointNet uses a 2D detection frame to generate a 3D cone to realize cross-modal sparse point cloud processing. Its cone space compression technology reduces the calculation amount of the global point cloud search range by 90%, and the end-to-end original point cloud processing architecture improves the detection accuracy of small targets by 12.3% (on KITTI dataset [76]), and the cross-modal weak supervised learning uses 2D tags to guide 3D training to accelerate convergence. However, this method relies on rigid geometric transformation, and the error of dynamic scene is up to 15–20%, and the performance drops sharply in a low-light environment. This method can be improved in many aspects. For example, F-ConvNet [77] uses neighborhood feature embedding to enhance the robustness of dark light. The innovation of F-PointNet is that it verifies the feasibility of “2D prior with sparse point cloud” for the first time, laying the foundation for MV3D [78], PointPiers [79] and other subsequent multimodal fusion and efficient encoder design.

FSF: FSF integrates 2D instance segmentation with LiDAR point cloud processing to achieve fully sparse cross-modal fusion. Its core contribution lies in instance-level sparse fusion, which significantly improves the inference speed. Additionally, FSF introduces a dual-modal instance generation module and a prediction module to enhance the detection accuracy. By fusing the high semantic resolution of 2D images with the geometric precision of LiDAR, FSF significantly improves the robustness in terms of detecting small objects and dynamic scenes. However, since FSF relies primarily on the camera branch for fusion, it underutilizes LiDAR data, leading to reduced accuracy in geometry-sensitive scenarios and degraded performance under low-light or adverse weather conditions (e.g., rain, fog). Future work could explore temporal fusion and modality-balancing mechanisms for extreme environments to further enhance the robustness.

SparseFusion: SparseFusion significantly improves the efficiency and performance of long-distance perception through a completely sparse representation method. It realizes the end-to-end process from sparse candidate generation to sparse fusion for the first time, avoiding the computational redundancy of traditional dense BEV features. The core innovation lies in the design of a semantic–geometric cross-modal transfer module, which achieves modality interaction through a lightweight self-attention mechanism while incorporating geometric alignment and semantic compensation strategies, effectively addressing the semantic deficiency of sparse point clouds in long-range scenarios. In addition, like FSF, SparseFusion adopts a two-stage label allocation and shape alignment strategy to solve the problem of the insufficient recall rate of the LiDAR-only method on low-cloud-density targets. This method improves the AP of small target detection by 8.2% (nuScenes dataset) and is more robust to sensor calibration errors than BEVFusion and other dense fusion methods. However, due to the over-dependence of this method on the camera mode rather than LiDAR, the reliability of the semantic information of the camera branches decreases in long-distance or low-light conditions (such as night, rain and fog), resulting in the degradation of performance. The subsequent improved method, such as SparseLIF [80], optimizes the modal equilibrium through uncertain perceptual fusion, but the problem of performance degradation in extreme environments is not completely solved.

MV2DFusion: MV2DFusion achieves a breakthrough in the field of multimodal detection through the modal-balanced sparse fusion architecture and dynamic query generation mechanism. It designs a double branch query generator, in which the image query uses the probability distribution to model the depth uncertainty, and the point cloud query uses the voxel features to retain the geometric prior, and the two achieve feature complementarity through the attention mechanism, reaching 76.7% NDS on the nuScenes dataset. Its target-level semantic fusion paradigm dynamically balances the modal weights, which solves the problem of modal bias dominated by camera or LiDAR in traditional methods. It supports 2D/3D detector plugging, and the reasoning speed is up to 5.5 FPS. Experiments show that although the time-series modeling ability of this method needs to be improved in extreme motion scenes (such as sudden braking vehicles or suddenly turning pedestrians), resulting in a relative lack of robustness, its multimodal fusion performance is excellent in normal dynamic scenes and static scenes, showing excellent scene adaptability. It is worth noting that the framework can maintain good real-time performance while ensuring high detection accuracy, which demonstrates its feasibility for practical application. In the future, the performance may be further improved by a timing memory module or edge optimization technology.

TransFusion: The core innovation of TransFusion lies in its dynamic feature–proposal interaction mechanism to address cross-modal misalignment. By leveraging Transformer-based cross-attention, the framework adaptively aligns sparse LiDAR proposals with dense camera features for dynamic multimodal fusion. While sparse feature processing ensures computational efficiency, its quadratic complexity remains a limitation—compared to SparseFusion memory savings, TransFusion prioritizes inter-modal interaction quality over sparsity optimization. Similar to SparseFusion, they both have camera dependency. Future work could integrate SparseFusion’s sparse computational efficiency with TransFusion’s dynamic alignment capability to enhance the robustness of multimodal perception.

Table 3 summarizes the advantages and limitations of the decision-level fusion algorithms. The analysis reveals that decision-level fusion offers the following three primary advantages. (1) High flexibility: It enables the application of distinct processing algorithms for each modality, demonstrating strong adaptability to various data characteristics. (2) Implementation simplicity: Compared to feature-level fusion, decision-level fusion typically presents lower implementation complexity as it primarily handles the final outputs rather than intermediate features. (3) Enhanced interpretability: The independent outputs from each modality facilitate comprehensive analysis and understanding of individual modality contributions to the final decision-making process. However, this approach also exhibits several limitations. (1) Information loss: The independent processing of modalities prior to fusion may lead to partial information degradation, particularly in complex scenarios where inter-modal correlations are crucial. (2) Performance dependency on individual modalities: The overall detection performance may be significantly compromised if any single modality underperforms. (3) Fusion strategy complexity: The selection of appropriate fusion strategies requires careful consideration of specific application contexts, thereby increasing the design complexity. Current research directions for improving decision-level fusion primarily focus on four aspects: dynamic weight allocation, ensemble learning, interpretability enhancement, and post-processing optimization. These improvements aim to enhance the accuracy, robustness, and flexibility of decision-level fusion, thereby better addressing the requirements of practical applications. At the same time, we must consider the real-time performance of the model, which is a major factor in its ability to be implemented and put into use [81,82,83]. This is also a necessary condition for ensuring the safety of autonomous driving [84].

We divide feature fusion into feature-level fusion and proposal-level fusion according to the abstract level and information processing method of fusion. The former pursues the joint representation of the original data, and the latter focuses on the collaborative decision-making of high-level reasoning. When the task depends on fine-grained information (such as pose estimation) and modal alignment is feasible, feature-level fusion has more obvious advantages, while when the modal differences are large (such as text with image) or fault tolerance is required (such as sensor redundancy), proposal-level fusion can give full play to its advantages. In fact, in real systems, the two are often combined at the hierarchical level to give consideration to accuracy and robustness. This not only refers to the field of automatic driving but also includes the field of medical AI and so on.

**Figure 3:**
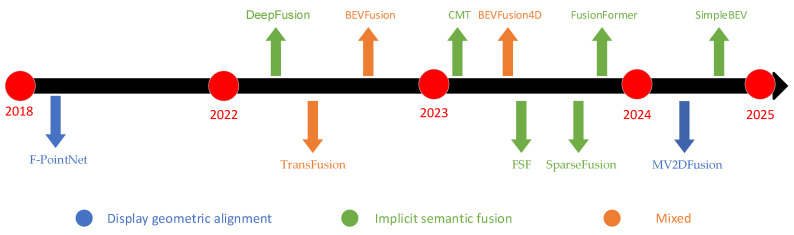
Chronological evolution of multimodal learning methods based on explicit geometric alignment and implicit semantic fusion.

In addition, we propose a novel taxonomy of the above-mentioned literature based on the explicit geometric alignment, the implicit semantic fusion, and their hybrid strategies, with a chronological evolution map illustrated in Figure 3. By comparison, it is found that in the field of feature fusion, the trend is generally shifting from early explicit alignment (such as F-PointNet) to implicit fusion (such as Transformer-based methods), which once again reflects the advantages of the Transformer architecture and end-to-end learning, and also corresponds to the current trend of hot large-scale models. But will displaying alignment really lead to elimination? Obviously, it is not like that. Compared with implicit semantic fusion methods, display alignment has advantages such as high spatial accuracy, good interpretability, and good real-time performance. However, it also has disadvantages such as scene limitations, dependence on feature quality, and insufficient flexibility. One major reason for the trend of feature fusion shifting from display alignment to implicit semantic fusion is the semantic robustness and end-to-end learning of implicit semantics, which can help models better adapt to polysemy, multimodal discrimination, and sparse data modality complementarity. The BEVFusion series introduces implicit semantic fusion while retaining display alignment, aiming to balance accuracy and flexibility. Although it has achieved great success, there are still issues with low real-time performance.

The BEVFusion series introduces implicit semantic fusion while retaining display alignment, aiming to balance accuracy and flexibility. Although it has achieved great success, there are still issues with low real-time performance, poor robustness in extreme environments and so on. In the future, implicit semantic fusion structures will shine brightly in the field of feature fusion, such as SparseFusion and SimpleBEV, which will further optimize the efficiency and sparsity of implicit fusion. However, the explicit geometric alignment method will definitely not be discarded. The huge success achieved by MV2DFusion further confirms that adding a part of the display alignment method to implicit semantic fusion is a better solution to the problem.

## 5. Datasets and Evaluation Metrics

### 5.1. Datasets

A dataset is a curated collection of data within a specific research or application domain, primarily used for training, validating, and testing machine learning models or conducting data analysis [85]. As such, datasets typically include subsets such as training sets, test sets, and validation sets. Serving as the foundation of object detection systems, the quality and diversity of datasets directly influence the model performance and application effectiveness, making the selection of an appropriate dataset critical [86].

The impact of datasets on object detection systems manifests in several key areas. For model training, datasets provide the necessary samples to train machine learning or deep learning models. High-quality and diverse datasets enable models to learn more discriminative features, thereby enhancing the object detection accuracy. For validation and testing, datasets allow for the evaluation of model performance on unseen data, ensuring robust generalization capabilities [87]. In terms of annotation, the accuracy of the labels in a dataset is fundamental to model learning, as precise annotations significantly improve model performance. Regarding diversity and representativeness, datasets encompassing varied environments, lighting conditions, perspectives, and object categories help models adapt to real-world scenarios, improving their robustness. Data augmentation techniques, such as rotation, scaling, and flipping, can further expand datasets, increasing the sample diversity and mitigating overfitting. For benchmarking, publicly available datasets are often used as standards to compare the performance of different algorithms, driving advancements in the field [88]. In transfer learning, when annotated data are insufficient to train a robust object detection model, models pre-trained on large-scale datasets can be fine-tuned for specific tasks, leveraging the knowledge from broader datasets.

Datasets can be broadly categorized into large-scale, publicly available ones, such as KITTI [76], nuScenes [67], and Waymo [89], and smaller, specialized datasets that are often unpublished [90]. Examples of the latter include datasets focused on rainy road conditions [91] or lotus root semantic segmentation [92]. These smaller datasets are typically developed by individuals or teams for specific applications and often yield superior results for targeted tasks. Table 4 provides a summary of the commonly used object detection datasets.

### 5.2. Evaluation Metrics

Evaluation metrics serve as essential criteria for assessing the performance of object detection algorithms. These metrics are generally divided into two categories: those evaluating the classification capability of the algorithm, such as the accuracy, precision, error rate, recall, precision–recall (PR) curves, receiver operating characteristic curves (ROC), average precision (AP), and mean average precision (mAP), and those focusing on the localization accuracy of detected targets, such as intersection over union (IoU).

**Figure 4:**
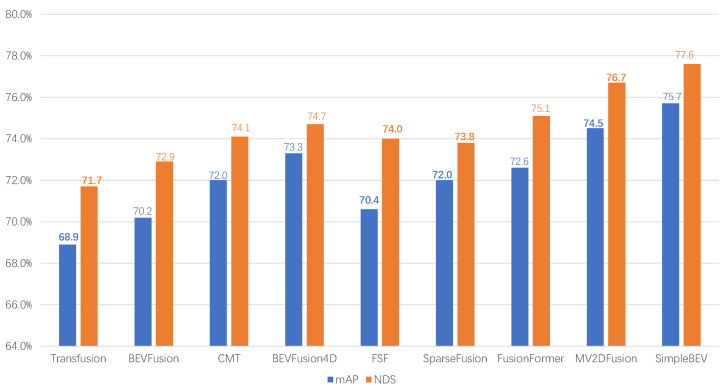
Comparative performance evaluation on the nuScenes dataset.

IoU quantifies the accuracy of spatial feature prediction in object detection by measuring the overlap between the predicted bounding box and the ground truth bounding box. A higher IoU indicates better localization accuracy, though it may also result in a higher miss rate. Precision is defined as the proportion of correctly predicted targets among all the predicted targets, while recall represents the proportion of correctly predicted targets among all the ground truth targets. The PR curve, plotted based on the precision–recall values, provides a comprehensive evaluation of the detector’s performance. The ROC curve, commonly used to assess the predictive accuracy of a model, indicates better performance as the curve deviates further from the baseline. The AP, calculated as the area under the PR curve, reflects the classifier’s performance, with higher AP values indicating superior results. For multi-class object detection, the mAP is used, which is the average of the AP values across all categories. Higher precision and recall values correspond to more accurate and reliable object detection results. The APH is an improved 3D object detection evaluation metric that adds a weighting of the heading accuracy to the standard AP. The relevant formulas are defined as follows:

(1) $${Precision}_{ij}=\frac{{TP}_{ij}}{{TP}_{ij}+{FP}_{ij}}$$

(2) $${Recall}_{ij}=\frac{{TP}_{ij}}{{TP}_{ij}+{FN}_{ij}}$$

(3) $${AP}_{i}=\frac{1}{m}\sum_{j=1}^{m}{Precision}_{ij}$$

(4) $$mAP=\frac{1}{n}\sum_{i=1}^{n}{AP}_{i}$$

(5) $$APH=\int_{0}^{1}{Precision}_{heading-weight}(Recall)dRecall$$

where ${Precision}_{ij}$ represents the precision of class i in the j-th image, and ${Recall}_{ij}$ denotes the recall of class i in the j-th image. TP (true positive) refers to correctly predicted targets, while FP (false positive) indicates targets that were not detected. A higher recall rate implies that the model detects more targets. ${AP}_{i}$ represents the average precision for class i. And mAP (mean average precision) is the average of the ${AP}_{i}$ values across all target categories. Table 5 presents a performance comparison between the feature-level feature fusion algorithms and proposition-level feature fusion algorithms on the nuScenes dataset. The evaluation is based on the metrics provided by the dataset, namely the mean average precision (mAP) and the nuScenes detection score (NDS). To better illustrate the performance of different methods on the nuScenes dataset, we also plotted a bar chart, as shown in Figure 4.

Based on the analysis of the bar charts and tables, as well as current research trends, we believe that the current development trend in the field of multimodal feature fusion presents a trend of mutual reference and collaborative evolution between explicit geometric alignment and implicit semantic fusion. From the perspective of time evolution, early explicit alignment methods represented by F-PointNet dominated with spatial accuracy and interpretability, while with the popularity of the Transformer architecture, implicit fusion has become the mainstream trend, with the advantages of end-to-end learning and semantic robustness. However, explicit alignment has not been eliminated—its advantages in terms of real-time performance, spatial positioning accuracy, and other aspects make it irreplaceable in specific scenarios, such as the BEVFusion series in autonomous driving. The complementarity between the two is becoming increasingly prominent: implicit fusion can solve complex problems such as polysemy and modal complementarity, while explicit alignment can provide structured priors and interpretable paths. The future development direction will emphasize the organic integration of the two; for example, MV2DFusion embeds explicit alignment modules into implicit frameworks, retaining geometric accuracy while enhancing semantic adaptability. This evolutionary path indicates that the optimal solution is not a dichotomous choice but a dynamic balance between the explicit alignment of “hard constraints” and the implicit fusion of “soft adaptation” achieved through architectural innovation, driving feature fusion technology toward higher accuracy, stronger generalization, and better real-time performance.

## 6. Multi-Task Applications of Multi-Sensor Fusion Target Detection Algorithms

In the field of autonomous driving, multi-sensor fusion target algorithms are not only applied in object detection but also have significant applications in segmentation, tracking and path planning, lane detection, traffic flow analysis, and dynamic scene understanding. Table 6 lists the application scenarios of multi-sensor target detection algorithms in various contexts.

### 6.1. Segmentation

In the task of segmentation using multi-sensor target detection algorithms, it is essential not only to accurately identify the boundaries of known objects but also to effectively process data from different sensors to enhance the segmentation precision. Segmentation not only finds applications in the realm of autonomous driving but also plays a significant role in agriculture and unmanned aerial vehicles (UAVs), among others [102]. For instance, the segmentation of fruits and wheat is employed to achieve precision smart agriculture [103,104,105]. Zhuang et al. [106] proposed a perception-aware multi-sensor fusion (PMF) collaborative fusion scheme to leverage perceptual information from two modalities. Additionally, they introduced an extra perceptual loss to measure the perceptual differences between the two modalities. Extensive experiments on datasets have demonstrated the superiority of their method. However, there are also problems such as insufficient feature extraction of sparse point clouds, low computational efficiency, poor real-time performance, and inherent errors in cross-modal alignment. These can be improved through methods such as sparse feature enhancement and lightweight design in the future.

### 6.2. Tracking and Path Planning

Tracking refers to the process of continuously detecting and identifying specific targets (such as pedestrians, vehicles, crops, or other obstacles) in dynamic environments [107]. It involves acquiring data from sensors and updating the target’s position and state in real time. Path planning, on the other hand, refers to the process of calculating an optimal or feasible driving route for autonomous vehicles in a known environment, the realization of which is inseparable from the implementation of tracking. Ye et al. [108] designed a Transformer-based fusion architecture, FusionAD, on the basis of BEV perception, which integrates temporal and contextual information to fuse, train, and summarize information from multiple modalities in three stages, significantly enhancing the model’s capabilities in terms of tracking and prediction. Further improvements can be made to FusionAD through dynamic calibration, lightweight computation, and open environment generalization. Moreover, tracking has numerous applications in agriculture, such as using tracking technology to address the automatic navigation of unmanned agricultural tractors [109], setting wheat harvest boundary lines for autonomous combine harvester path tracking [110], and utilizing tracking technology to control the path of unmanned rice transplanters for rice planting operations [111].

### 6.3. Lane Detection

Lane detection is one of the fundamental components essential for achieving fully autonomous driving [112]. Accurate lane information is critical for vehicles to make autonomous decisions under various driving conditions. Lane detection not only serves as a basic element for ensuring safety but also acts as a crucial foundation for efficient navigation and intelligent decision-making [113,114]. Furthermore, lane detection can be extended to agricultural applications, such as detecting furrows in fields [115], laying the groundwork for future smart agriculture. Zhang et al. [116] proposed a novel multimodal lane detection model, termed Multi-Modal Attention-Guided Real-Time Lane Detection, which integrates an attention mechanism into the network to balance the multimodal feature fusion and enhance the detection capabilities. Subsequent extensive experiments were conducted on the processed sequential KITTI dataset, and the results demonstrated that the attention mechanism significantly improved the performance of multimodal detection by effectively balancing multimodal features. This work provides a scalable attention fusion paradigm for multimodal lane detection, and in the future, research can further overcome the practical deployment bottlenecks by combining lightweight design and cross-modal generalization.

### 6.4. Traffic Flow Analysis

Traffic flow analysis plays a pivotal role in comprehending the surrounding environment and discerning the behaviors of other traffic participants, thereby facilitating informed driving decisions. Multi-sensor fusion target detection algorithms are capable of acquiring information such as traffic lights or road signs, as well as detecting obstructing objects, enabling more judicious decision-making. Li et al. [117] proposed a novel model, MDCGCN, which comprises three major components, each consisting of two sub-components: a baseline adaptation mechanism and a multi-sensor correlation convolutional block. These sub-components not only mitigate discrepancies in periodic data, thereby enhancing the quality of the data input, but also effectively capture dynamic spatiotemporal correlations induced by changes in inter-road traffic patterns. Extensive experiments have demonstrated that this model significantly improves the accuracy of medium- and long-term traffic predictions across various scales of traffic networks. However, due to the computational efficiency bottlenecks, it is difficult to meet real-time requirements. In the future, lightweighting and continuous modeling can be used to further overcome the practical deployment bottlenecks.

### 6.5. Dynamic Scene Understanding

Dynamic scene understanding aims to analyze and interpret objects, events, and interactions in real time within continuously changing environments. This involves processing video streams or sequential image frames to identify, track, and predict the behaviors and states of objects in the scene. By recognizing user actions and intentions, dynamic scene understanding plays a critical role in enabling autonomous vehicle navigation. Multimodal data fusion can provide richer information, enhancing the accuracy and comprehensiveness of the scene understanding [118]. Zhu et al. [119] proposed a multi-sensor signal fusion method based on PV-RCNN and LapDepth (PV-LaP) to improve the 3D visual scene understanding. By integrating camera and LiDAR data, the PV-LaP method enhances the accuracy of the environmental perception. Evaluated on the KITTI datasets, the PV-LaP framework demonstrates superior performance. In addition to the field of autonomous driving, it also has significant value in areas such as robot visual servoing, augmented reality (AR), and smart city monitoring.

## 7. Summary and Outlook

This article provides a comprehensive overview of the evolution of classical and state-of-the-art algorithms in the field of multi-sensor fusion-based object detection, categorizing them into feature-level and decision-level fusion approaches and systematically analyzing their respective strengths and limitations. Feature-level fusion achieves efficient alignment of multimodal data through a unified representation space (such as BEV), but the computational complexity is relatively high. Proposal-level fusion is based on high-level semantic interaction, with greater flexibility, but it heavily relies on unimodal performance. The current research trend shows that the Transformer architecture significantly improves the cross-modal fusion capability through the self-attention mechanism, while sparse design (such as SparseFusion) and temporal modeling (such as BEVFusion4D) further optimize the real-time performance and dynamic scene adaptability. However, there are still issues such as poor robustness in extreme environments, low computational efficiency, and imbalanced modes. Future research directions include lightweighting, adaptive fusion, and cross-modal pre training. Moreover, we introduce relevant datasets and evaluation metrics while emphasizing the significant applications of multi-sensor fusion object detection algorithms. With the continuous advancement of multi-sensor fusion technology, the emergence of novel frameworks, and the development of new tasks, these algorithms are expected to become increasingly sophisticated, achieving higher accuracy and enabling more robust multi-task capabilities, ultimately contributing to the realization of fully autonomous driving.

As technology progresses and application demands grow, the future development prospects for multi-sensor fusion object detection algorithms are highly promising. Future research will focus on exploring advanced data fusion techniques, such as deep learning and graph neural networks, to enhance the effectiveness of integrating diverse sensor data. The development of adaptive learning mechanisms will enable systems to update and adjust models in real time, ensuring adaptability to new environments and objects. Algorithm optimization will prioritize low-latency processing and reduce computational resource consumption for real-time applications. Additionally, research will aim to improve the cross-domain transfer capabilities, allowing trained models to be effectively applied across different environments and tasks. The integration of multimodal learning approaches will expand beyond traditional visual and LiDAR data to incorporate other sensor types. At the same time, innovative ideas from other specialized methods such as real-time mapping and medical imaging, such as adaptive projection and diffusion models, can be considered for addition to object detection to increase its accuracy and real-time performance. In addition, how to achieve higher real-time performance while maintaining high accuracy is a key research focus, as improving accuracy often means an increase in computational complexity and a decrease in computation time. Finding a better balance between the two is also a major challenge.

The future of multi-sensor object detection algorithms is filled with opportunities, not only in autonomous driving but also in diverse fields such as agriculture and unmanned aerial vehicles [120]. Through continuous technological innovation and application expansion, these algorithms will play an increasingly critical role in complex environments. As research progresses, more efficient, intelligent, and versatile multi-sensor object detection solutions are expected to emerge, driving advancements across multiple domains.

## Figures and Tables

**Table 1:** Comparative characteristics of key sensors in autonomous vehicles.

Characteristic	Camera	LiDAR	Millimeter-Wave Radar
Data Type	2D RGB/grayscale images	3D point clouds	1D/2D range-velocity
Resolution	High (texture/color)	High (spatial)	Low (spatial), high (velocity)
Detection Range	Tens to hundreds of meters	Tens to hundreds of meters	Hundreds of meters
Environmental Robustness	Lighting-dependent	Lighting-insensitive	All-weather robustness
Output Information	Semantic features	Distance and shape data	Distance and velocity data
Advantages	Cost-effective, rich texture details	High-precision ranging	All-weather capability, superior speed measurement
Limitations	Lighting-sensitive, no direct ranging	High cost, large data volume	Low spatial resolution, limited geometric details

**Table 2:** Overall analysis of the feature-level fusion algorithms.

Algorithm	Advantages	Limitations
DeepFusion	Effectively integrates data from different sensors, improving accuracy.	High computational complexity, potentially compromising real-time performance.
BEVFusion	Provides more intuitive spatial information, aiding in complex scene processing.	High dependency on sensor position and orientation, potentially affecting fusion quality.
BEVFusion4D	Captures temporal changes, enhancing detection capability for dynamic objects.	Increased model complexity and higher computational resource requirements.
SimpleBEV	Simple structure, easy to implement and deploy.	Detection performance in complex scenes may lag behind more sophisticated models.
FusionFormer	Self-attention mechanism enhances global context modeling.	High computational overhead during training and inference, potentially impacting real-time applications.
CMT	Flexibly handles information interaction between different modalities.	High demands on model design and hyperparameter tuning, increasing complexity

**Table 3:** Overall analysis of the proposal-level fusion algorithms.

Algorithm	Advantages	Limitations
F-PointNet	Efficient point cloud processing with strong robustness.	High computational complexity may hinder real-time applications.
FSF	Adaptive feature selection enhances fusion effectiveness and flexibility.	Complex implementation of adaptive mechanisms depends on feature quality.
SparseFusion	Sparse data specialization reduces computational burden and improves efficiency.	Potential information loss in sparse scenarios may degrade fusion performance.
MV2DFusion	Effective multimodal integration enhances contextual understanding in dynamic scenes.	High computational demands due to multi-view processing and view dependency.
TransFusion	Enables robust occlusion handling via dynamic feature–proposal alignment in an end-to-end framework.	Transformer-based cross-attention scales quadratically with the number of proposals, posing challenges for deployment on edge devices.

**Table 4:** Commonly used object detection datasets.

Dataset Name	Total Images	Total Size	Label Categories
KITTI	15,000	1242 × 375	11
nuScenes	40,000	1600 × 900	10
Waymo Open Dataset	2,000,000	1280 × 720	6
ApolloScape [93]	20,000	1280 × 720	5
Cityscape [94]	5000	2048 × 1024	30
Oxford RobotCar [95]	2,000,000	1280 × 960	-
Argoverse2 [96]	500,000	1920 × 1200	10
PandaSet [97]	48,000	1920 × 1080	10
A2D2 [98]	246,000	1920 × 1080	10
H3D (Honda) [99]	100,000	1920 × 1080	5
Euro-PVI [100]	20,000	640 × 480	2
KAIST Multi-Spectral [101]	50,000	640 × 480	2

**Table 5:** Performance comparison of feature-level fusion algorithms and proposal-level fusion algorithms on the nuScenes dataset.

Type	Model	mAP	NDS
Feature-Level Fusion Methods	BEVFusion	70.2%	72.9%
	BEVFusion4D	73.3%	74.7%
	SimpleBEV	75.7%	77.6%
	FusionFormer	72.6%	75.1%
	CMT	72.0%	74.1%
Proposal-Level Fusion Methods	FSF	70.4%	74.0%
	SparseFusion	72.0%	73.8%
	MV2DFusion	74.5%	76.7%
	Transfusion	68.9%	71.7%

**Table 6:** Applications of multi-sensor object detection algorithms.

Related Literature	Application Domain	Application of Multi-Sensor Target Detection Algorithms
Perception-Aware Multi-Sensor Fusion for 3D LiDAR Semantic Segmentation.	Segmentation	Proposed a network incorporating two workflows (LiDAR and camera) to leverage information from both modalities.
FusionAD: Multimodality Fusion for Prediction and Planning Tasks of Autonomous Driving.	Tracking and Path Planning	Constructed a Transformer-based multimodal fusion network to effectively generate fusion-based features.
Multi-Modal Attention-Guided Real-Time Lane Detection.	Lane Detection	Utilized multi-frame input and long short-term memory networks to address vehicle occlusion, lane line detection, and marking degradation issues.
Traffic Flow Prediction Over Multi-Sensor Data Correlation with Graph Convolution Network.	Traffic Flow Analysis	Introduced a novel model capable of eliminating differences in periodic data while effectively capturing inter-traffic pattern relationships.
PV-LaP: Multi-Sensor Fusion for 3D Scene Understanding in Intelligent Transportation Systems.	Dynamic Scene Understanding	Enhanced environmental perception accuracy by integrating camera and LiDAR data.
